# Case report: Exploring chemoradiotherapy-induced leukoencephalopathy with 7T imaging and quantitative susceptibility mapping

**DOI:** 10.3389/fneur.2024.1362704

**Published:** 2024-02-14

**Authors:** Gaetano Celardo, Elena Scaffei, Bianca Buchignani, Graziella Donatelli, Mauro Costagli, Paola Cristofani, Raffaello Canapicchi, Rosa Pasquariello, Michela Tosetti, Roberta Battini, Laura Biagi

**Affiliations:** ^1^Department of Developmental Neuroscience, IRCCS Stella Maris Foundation, Pisa, Italy; ^2^Department of Clinical and Experimental Medicine, University of Pisa, Pisa, Italy; ^3^Department of Translational Research and of New Surgical and Medical Technologies Pisa University, Pisa, Italy; ^4^Imago 7 Research Foundation, Pisa, Italy; ^5^Department of Neuroscience, Rehabilitation, Ophthalmology, Genetics, Maternal and Child Health (DINOGMI), University of Genoa, Genoa, Italy

**Keywords:** leukoencephalopathy, ultra-high field MRI, 7 Tesla, neurotoxicity, chemoradiotherapy, brain damage

## Abstract

Chemotherapy and radiotherapy are widely used in the treatment of central nervous system tumors and acute lymphocytic leukemia even in the pediatric population. However, such treatments run the risk of a broad spectrum of cognitive and neurological deficits. Even though the correlation with cognitive decline is still not clear, neuroradiological defects linked to white matter injury and vasculopathies may be identified. Thanks to the use of 7T MRI it is possible to better define the vascular pattern of the brain lesions with the added advantage of identifying their characteristics and anatomical localization, which, however, are not evident with a conventional brain scan. Moreover, the use of Quantitative Susceptibility Mapping (QSM) makes it possible to discriminate between calcium deposits on vessels (chemo-radiation-induced) and hemoglobin deposition in radio-induced cavernomas, speculating, as a result, about the pathophysiology of iatrogenic brain damage. We describe the case of a 9 year-old boy with a T-type acute lymphoid leukemia who had previously been treated with polychemotherapy and high-dose RT. To better define the child's neuroradiological pattern, 7T MRI and QSM were performed in addition to conventional imaging examinations. Our case report suggests the potential usefulness of a QSM study to distinguish radio-induced vascular malformations from mineralizing microangiopathy.

## Introduction

The effectiveness of therapy in the onco-hematologic field has markedly improved over the past decades, with the childhood cancer survival rate reaching approximately 80% at 5 years from diagnosis (1). This rapid expansion of treatment options and the parallel growth in survival rate call for urgent clinical attention regarding the spectrum of both acute and chronic sequelae due to cancer treatment, including neurocognitive and psychiatric outcomes, as these features can greatly influence the children quality of life (2). Neurobehavioral morbidity in childhood cancer survivors affects several aspects of cognitive function, which can include attention, memory, processing speed, intellect, academic achievement, and emotional health. Neurological sequelae are a frequent side effect of many chemotherapy (CT) protocols, particularly when agents are given at high doses or administered intrathecally (2, 3), and when combined with cranial radiation therapy (RT).

The developing central nervous system (CNS) brain of a child is particularly susceptible to the adverse effects of cancer treatment, which not only causes brain injury, but also alters crucial developmental events, such as myelination, synaptogenesis, neurogenesis, cortical thinning, and the formation of neuronal networks leading to complex neurobehavioral morbidity [for an extensive and updated review on clinical reports on the field see (4)].

More in detail, RT complications are often divided into short-term and long-term adverse effects. Acute adverse effects generally occur within 6 weeks of RT and are often self-limited. Late or delayed complications of cranial RT can occur months or years after treatment and are often irreversible, including cerebrovascular effects, radionecrosis and vasculopathies (5). The pathophysiology of RT-induced brain damage still remains largely unclear (6). Over the past two decades research has highlighted that the cellular response to radiation injury in the brain involves multiple cell types including astrocytes, microglia, oligodendrocytes, endothelial cells, and neurons that initiate and respond to inflammatory cascades and contribute to progressive neurological damage (7).

However, even CT may be highly toxic for the pediatric brain (8). In particular, methotrexate (MTX) is known for its significant adverse effects on the CNS, with concomitant brain radiation and young age identified as risk factors for MTX-associated issues (9). Although the majority of chemotherapy agents does not cross the intact blood brain barrier (BBB), the amount of neurotoxicity produced by a multimodal therapy regimen potentially reduces the integrity of the barrier. Chemotherapy can also lead to apoptosis and, in addition, can impair neurogenesis and worsen neurocognitive functions through DNA damage and inflammation, thus accelerating neural aging in survivors (10).

Neuroimaging abnormalities have been reported in several studies following CT, RT, or multimodal therapy. These abnormalities encompass common conditions such as leukoencephalopathy (LE) and vasculopathies, microbleeds and cavernomas, but also more rarely observed cyst-like lesions (CLL) (9, 11). Children who receive RT at younger ages may be especially vulnerable to radiation-induced LE (4) and the risk of LE is correlated with the dose and volume of the brain which has been exposed to radiation. Radiation exposure to the whole brain at doses >24 Gy increases the risk of LE (12, 13), but LE has also been identified when doses of 18 Gy are combined with high-dose intra-venous or intrathecal MTX (4, 14).

Neuroimaging at 7 Tesla provides superior signal-to-noise and contrast to-noise ratios, as well as an increased spatial resolution compared to high filed MRI, thus allowing an increased conspicuity and spatial characterization of brain lesions (15). Furthermore, sensitivity to susceptibility phenomena increases with increasing magnetic field strength, enhancing the tissue contrast in susceptibility-weighted images. Trade-offs include greater safety concerns such as a higher specific absorption rate, but also methodological constraints such as longer scanning times and increased distortion and field inhomogeneity. Quantitative susceptibility mapping (QSM) is an MR technique which is able to measure the magnetic susceptibility (χ) of human tissues (16) and estimate brain iron deposition (17). From a clinical research perspective, QSM could be useful to quantitatively estimate the susceptibility of brain lesions, to differentiate between paramagnetic (e.g., non-heme iron and hemosiderin) and diamagnetic (e.g., calcium) components, and measure possible susceptibility changes over time, by leveraging on its remarkable reproducibility with clinically feasible protocols (18).

Despite the increasing use of 7T MRI for research purposes in different fields (19) reports of this technique in the developmental age remain sparse and are mostly focused on epilepsy (20, 21). According to CARE guidelines, here we report the boy with a T-type acute lymphoid leukemia. The patient had been treated with polychemotherapy and high-dose RT and had who performed a 7T MRI. We describe the long term neuroradiological sequelae both at conventional (1.5 T) and at 7T MRI with interesting insights into the pathophysiology of iatrogenic brain damage, thanks both to the better image contrast and to the use of QSM.

## Case description

A 6-year-old boy referring to the hospital for cough and bilateral cervical lymphadenopathies was diagnosed with acute lymphoid leukemia (T-cell type). A brain MRI and EEG assessments yielded normal results and CT was started with a prophase, involving prednisone at a dosage of 60 mg/m^2^. The induction phase 1a included dexamethasone at 10 mg/m^2^/day, vincristine at 1.5 mg/m^2^, daunorubicin at 30 mg/m^2^ for several days, PEG-asparaginase at 2500 IU/m^2^, and intrathecal Methotrexate (MTX) at 12 mg. The induction phase 1b continued with Cyclophosphamide, Cytarabine, MTX, and the consolidation therapy with the application of 6-Mercaptopurine and MTX. Following this, a maintenance therapy ensued with MTX at 20 mg/m^2^ once a week and 6-mercaptopurine at 50 mg/m^2^/day, with the dosages being adjusted based on the trajectory of blood count values. Prophylactic cranial RT for the risk of recurrence in the central nervous system was assessed, and initially planned with a dosage of 12 Gy. However, subsequent checks revealed that a supramaximal dosage at 36 Gy had been administered. CT lasted for ~2 years, during which the child experienced several bronchopneumonia episodes requiring hospitalization and targeted antibiotic therapies. If, on the one hand, the patient showed a significant improvement in his internal clinical conditions, on the other his parents noticed a gradual regression of the child's scholastic skills. The child's ability to write and read correctly deteriorated progressively and he exhibited difficulties in motor coordination.

Due to this regression, at the age of 9 years the boy was, for the first time, referred to the pediatric neurology section of IRCCS Stella Maris Foundation for a multidisciplinary evaluation. Clinical examination revealed no neurological focal signs, mild clumsiness and poorly organized ocular motility. The boy's motor skills, assessed by the Movement Assessment Battery for Children (mABC2) test, were deficient for the child's chronological age (Standard score = 3, corresponding to the 1st percentile). The cognitive level assessed through the standardized WISC-IV scale was mildly deficient with weaknesses in verbal auditory working memory and processing speed (Total Intelligence Quotient 70, Verbal Reasoning Percentile 85, Visual Reasoning Percentile at 86, Memory and Learning Percentile at 70 Executive Functioning Percentile at 62). Emotionally, the child exhibited vulnerability with anxiety, having avoidance behavior when faced with complex tasks. The EEG assessment was normal.

A brain 1.5 T MRI revealed an extensive pattern of chemo-radio-induced leukoencephalopathy. The images revealed areas of signal hyperintensity in long TR images and hypointensity in T1-weighted images in the periventricular, deep, and subcortical white matter within the fronto-temporo-parietal regions. Cystic formations were identified in the left fronto-basal, anterior temporo-basal, right fronto-lateral, and anterior right semioval center. 3D multi-echo gradient-recalled echo (SWAN) imaging displayed numerous areas of signal hypointensity, which were primarily located in the juxtacortical and cortical fronto-temporo-parietal regions. Numerous areas of hypointensity were also observed in the lower cerebellar hemisphere. These findings were attributed to calcifications and deposits of hemosiderin. Additionally, in the same sequence, other areas of hypointensity were observed in the bi-pallidal region, which were likely linked to micro-mineralization phenomena. In the sub-tentorial region, a focal area of signal hyperintensity in the T2 weighted FLAIR images was observed in the left cerebellar hemisphere white matter, devoid of proton diffusivity restriction and signs of mass. The sella turcica appeared partially empty. T1 hyperintensity of the signal was present in the basicranium and the first two cervical metameres, suggesting fatty marrow conversion due to radiotherapy treatment.

**Figure 1:**
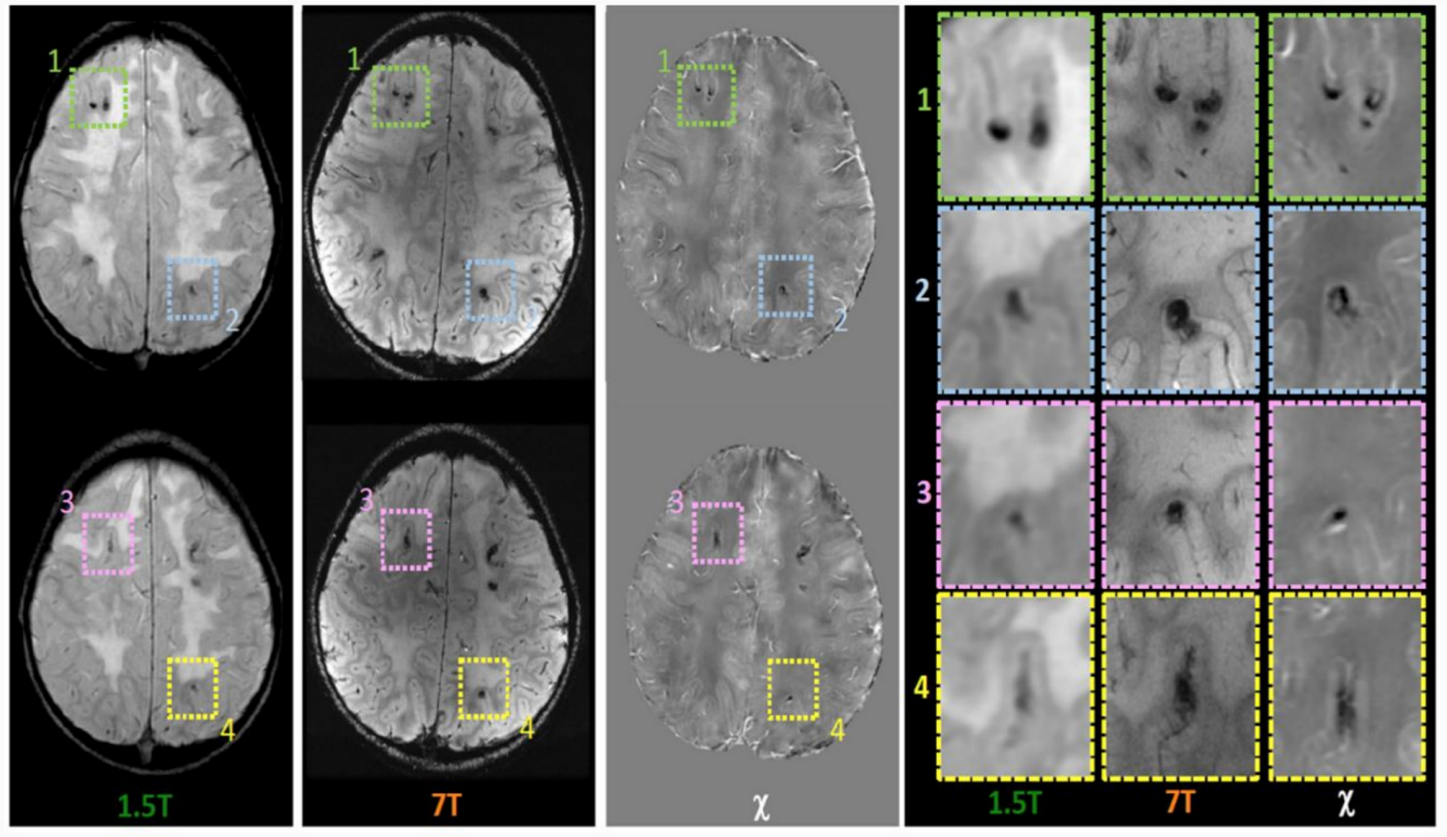
Magnetic susceptibility dependent axial images acquired at 1.5T and 7T and Quantitative Susceptibility (χ) Mapping analysis. While in the 1.5T images the foci of deposition (hypointense in all sequences) appear to be located at the cortico medullary junction, it is worth noting that in 7T images they appeared located in intracortical area as confirmed in images detail **(**panel on the **right)**.

**Figure 2:**
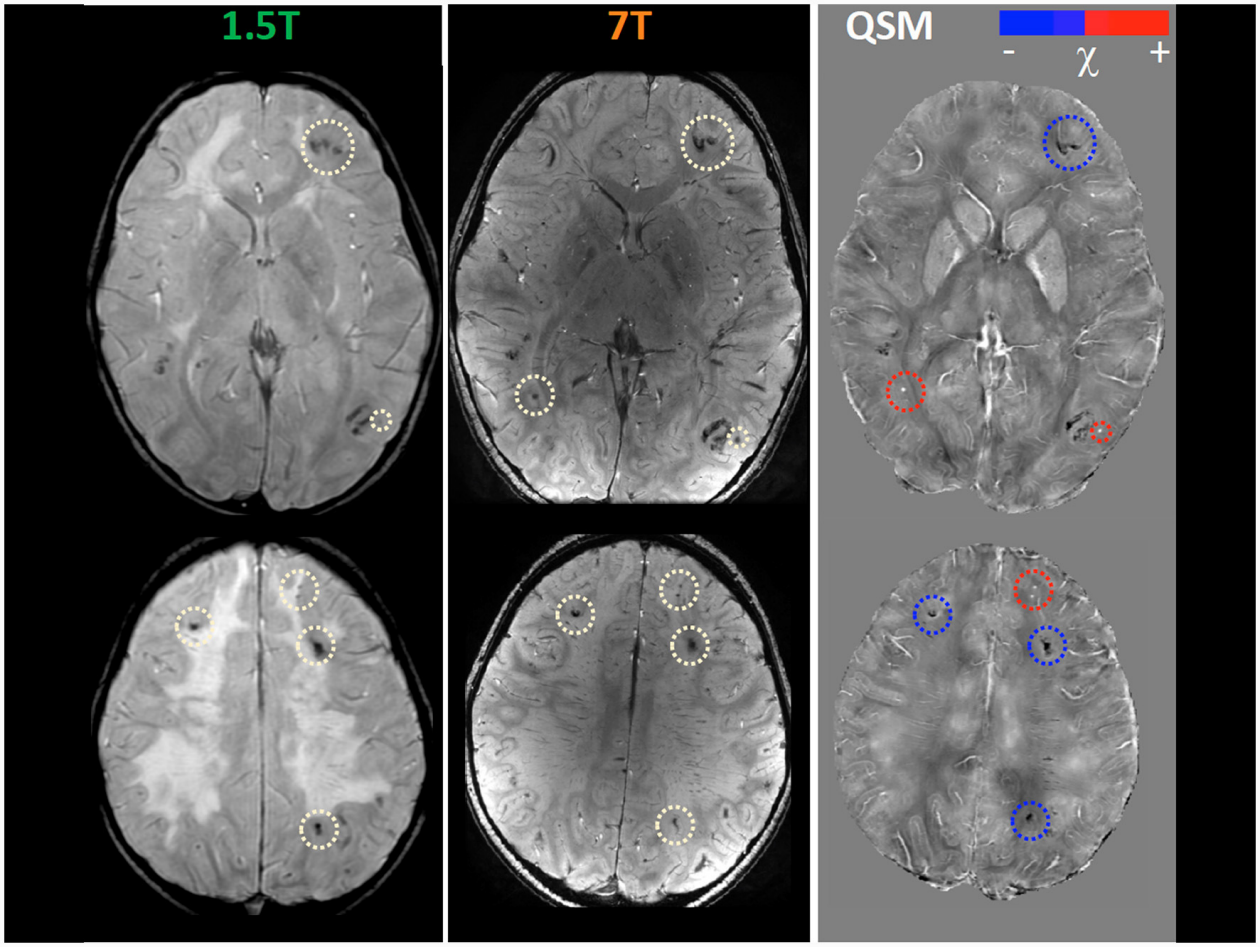
Magnetic susceptibility dependent sequence Quantitative Susceptibility (χ) Mapping (QSM) at 1.5T and 7T. Several focal hypointense formations are visible at the cortico-medullary junction in the two cerebral hemispheres (white circles), more numerous and evident in 7T images. Some of them (red circles) are hyperintense in the QSM images in in relation to the presence of material with high magnetic susceptibility, thus probably referring to radio-induced cryptic vascular malformations (cavernomas, capillary telangiectasias). Others (blue circles) remain hypointense in QSM images due to the prevailing presence of diamagnetic material, thus probably referring to parenchymal calcifications as a complication of multimodal chemo-radiotherapy treatment.

Within the research project BIANCA “Biomarker Imaging and New Challenging Approaches to assess white matter disorders in developmental age” a 7T brain MRI was also performed in order to better define brain lesion burden and etiology. The protocol included a 3D T1-weighted image (isotropic voxel size = 0.8 mm), a 2D Fast Spin Echo (FSE) T2-weighted sequence (in-plane resolution = 0.39 mm), a 3D T2-weighted Fluid Attenuated Inversion Recovery sequence (FLAIR; isotropic voxel size = 0.7 mm), and a 3D SWAN sequence tailored to obtain QSM (spatial resolution of reconstructed images = 0.35 x 0.35 x 0.7 mm^3^) following an established pipeline (22). T1-weighted and T2-weighted FLAIR images confirmed the findings described in 1.5 T MR exams, whereas, thanks to the higher spatial resolution and susceptibility-weighted contrast, FSE T2-weighted and susceptibility-weighted images improved the spatial localization of the T2^*^-hypointense signal alterations. Indeed, many lesions which appeared at the level of the corticomedullary junction in 1.5 T images were judged as intracortical in 7T images (Figure 1). Moreover, the dominant susceptibility source of the lesions was identified by using QSM, thus distinguishing small and round alterations with high susceptibility values, probably referring to radio-induced vascular malformations or microbleeds, in this case iuxtacortical, from intracortical diamagnetic alterations with a gyriform pattern, probably referring to mineralizing microangiopathies. The latter were probably caused by the combination of CT and RT treatment which caused calcium deposits and subsequently mineralizing microangiopathies (Figure 2).

## Discussion

We describe a boy with a complex iatrogenic leukoencephalopathy treated with multimodal, high-dose, CT and RT due to acute lymphoid leukemia (T-cell type), and we report neuroradiological findings both at conventional (1.5T) and ultra-high (7T) field MR images.

Our case report allowed us not only to confirm the recurrence of well-known symmetric and extensive Les in addition to neurodegeneration with calcium deposition in deep gray matter (GM), but also to shed light on the wide spectrum of vasculopathy induced by CT and RT. Vascular changes are often prominent in radiation-induced injuries and are likely to play an important role in its pathogenesis. Indeed, as one of the few actively proliferating sites in the brain, it is not surprising that the endothelial cell is particularly susceptible to radiation damage. The early toxic effect primarily impacts on BBB and is likely responsible for the vasogenic oedema seen in the acute or early delayed phases, albeit temporary and often steroid responsive (23). In contrast, more permanent forms of endothelial damage may account for the classic changes of chronic radiation injury, including thrombosis, hemorrhage, fibrinous exudates, telangiectasias, vascular fibrosis/hyalinization with luminal stenosis, and fibrinoid vascular necrosis. All of these changes facilitate hypoxic injury, white matter damage and parenchymal CNS necrosis (11).

To date several types of vasculopathy, involving both white and gray matter, are reported following RT and CT such as radiation vasculitis of the small brain vessels with hyalinization and fibrinoid necrosis of vascular walls, resulting in occlusion and infarction, as well as vascular proliferative lesions such as capillary telangiectasia and cavernoma, both leading to haemorrhagic lesions. Capillary telangiectasias usually occur 3–9 months after irradiation (24) while cavernomas take a longer time to develop after radiation therapy, possibily reflecting sequential variations of the same pathologic process (25). The unprecedented spatial resolution of images achievable with scanners operating at ultra-high magnetic field (≥7T) combined with the increased tissue contrast in T2^*^ weighted images improves the visibility of brain lesions and allows a more accurate morphological and spatial characterization. In our case report, high resolution 7T MR images revealed the intracortical localization of mineralizing microangiopathy and the iuxtacortical localization of paramagnetic vascular lesions/microbleeds. Interestingly, to date, only one other recent paper has studied post actinic leukoencephalopathy in a group of young adults with 7T MRI, focusing on cerebral microbleeds (26).

## Conclusions

Reports on chronic leukoencephalopathy among survivors of childhood cancer are continually increasing. In this case report we discuss the meaningful advantage of 7T MRI in the comprehension of the pathophysiology of chemo- and radiotherapy-induced brain damage. In particular, we highlight the potential value of QSM in dissecting the vascular contribution of white and gray matter injury. Indeed, QSM allows the clinician to distinguish radio-induced vascular malformations from mineralizing microangiopathy (a complication dependent on combined chemo-radio treatment). Moreover, even though lesions are classically described as affecting the basal ganglia (perforating vessels) and the cortico-medullary junction (arciform fibers) the use of 7T MRI proves that most lesions have an intracortical distribution. In conclusion, these data suggest the potential advantages of ultra-high field MRI in future longitudinal studies of chronic leukoencephalopathy among survivors of childhood cancer. Indeed, a better characterization of such a complex brain burden, which affects both the white and gray matter, could make a more precise monitoring of the neurobehavioural morbidity and its neuroanatomical correlates possible, which in turn would pave the way for new therapeutical interventions.

## Patient perspective

During all the clinical examinations and neuroimaging assessments, the patient and his parents were involved in supportive dialogues both with clinicians and with a clinician-trained psychologist. The patient managed to perform all the MRIs without the need of sedation.

## Data availability statement

The raw data supporting the conclusions of this article will be made available by the authors, without undue reservation.

## Ethics statement

The studies involving humans were approved by Comitato Etico Regione Toscana - Pediatrico. The studies were conducted in accordance with the local legislation and institutional requirements. Written informed consent for participation in this study was provided by the participants' legal guardians/next of kin. Written informed consent was obtained from the individual(s) for the publication of any potentially identifiable images or data included in this article.

## Author contributions

GC: Conceptualization, Data curation, Writing—original draft, Writing—review & editing. ES: Conceptualization, Supervision, Writing—original draft, Writing—review & editing. BB: Conceptualization, Supervision, Writing—original draft, Writing—review & editing. GD: Data curation, Methodology, Software, Writing—review & editing. MC: Data curation, Methodology, Software, Writing—review & editing. PC: Data curation, Writing—review & editing. RC: Data curation, Methodology, Writing—review & editing. RP: Data curation, Methodology, Software, Writing—review & editing. MT: Supervision, Validation, Writing—review & editing. RB: Conceptualization, Supervision, Writing—review & editing. LB: Conceptualization, Data curation, Methodology, Software, Supervision, Writing—review & editing.
